# Vesicular drug-delivery systems as theranostics in COVID-19

**DOI:** 10.4155/fmc-2020-0149

**Published:** 2020-06-26

**Authors:** Saurabh Satija, Meenu Mehta, Mousmee Sharma, Parteek Prasher, Gaurav Gupta, Dinesh K Chellappan, Kamal Dua

**Affiliations:** ^1^Discipline of Pharmacy, Graduate School of Health, University of Technology Sydney, Ultimo, NSW, 2007, Australia; ^2^School of Pharmaceutical Sciences, Lovely Professional University, Phagwara-144411, Punjab, India; ^3^Centre for Inflammation, Centenary Institute, Sydney, NSW, 2050, Australia; ^4^Department of Chemistry, Uttaranchal University, Dehradun, 248007, India; ^5^Department of Chemistry, University of Petroleum & Energy Studies, Dehradun, 248007, India; ^6^School of Pharmacy, Suresh Gyan Vihar University, Jagatpura, Mahal Road, Jaipur, India; ^7^Department of Life Sciences, School of Pharmacy, International Medical University, Bukit Jalil, 57000, Kuala Lumpur, Malaysia; ^8^School of Pharmaceutical Sciences, Shoolini University, Bajhol, Sultanpur, Solan, Himachal Pradesh, 173229, India; ^9^Priority Research Centre for Healthy Lungs, Hunter Medical Research Institute (HMRI) & School of Biomedical Sciences & Pharmacy, University of Newcastle, Callaghan, NSW, 2308, Australia

**Keywords:** COVID-19, theranostics, vesicular drug delivery

The World Health Organization (WHO) had announced the outbreak of coronavirus disease 2019 (COVID-19) as a pandemic on 11 March 2020. With the entire world reeling under the prodigious attack of the pandemic in collosal proportions, various strategies were designed to nullify the transmission, but with little success. Preventive measures, namely social distancing, sealing off large towns, closing borders and confining people to their homes were strategically implemented. Inspite of these, the transmission rates grew unabated. Adding fuel to fire, the lack of effective antiviral drugs and vaccine further worsened the scenario. Although, therapeutic strategies, namely repurposing drugs, adopting alternative or complementary systems and providing symptomatic treatment have, to a lesser extent helped, but the menace is largely rampant [1–3]. These factors have made it imperative for the urgent need of developing an effective ‘all-in-one package’ option having both diagnostics and therapeutics as a combined treatment approach. Such a theranostic approach could be a promising tool in combating the novel corona virus.

Theranostics is a diagnostic imaging technique that determines the target receptors that are present on a cell, and subsequently allows the receptors to be treated with an effective radiation therapy. In the antiviral theranostic approach, radiopharmaceuticals are largely used to identify and diagnose combination therapies that may prevent virus replication [4,5]. Recent data have revealed that theranostic drug therapies with chloroquine have effectively inhibited the 2019 novel coronavirus *in vitro*. Wang *et al.* tested the virus inhibition potential with another notable theranostic antiviral agent, penciclovir which demonstrated potent antiviral behavior. These findings have further strengthened and supported the need to develop newer technologies such as theranostics. This technology will also allow viral cell interaction monitoring and evaluation, employing clinical imaging when using its therapeutic potential alone or in combination with other antiviral drugs [6–8] as a co-delivery strategy. A study involving fast, responsive lateral flow immunoassay was conducted by Chen *et al.* to detect the anti-SRV-CoV-2 IgG in human serum using lanthanide-doped polysterene nanoparticles that could be further helpful in tracking the COVID-19 progression. Moreover, this assay will also help in assessing patient's treatment response [9].

Vesicular drug-delivery systems have been progressively employed as co-delivery tools for personalized theranostics that combine diagnostic, prognostic therapeutic and image-guided therapeutic effects [10]. Multifunctional- and multimodality-based theranostic techniques that employ vesicular drug-delivery systems are urgently needed to be developed for simultaneous imaging of the COVID-19 etiology. Vesicular-delivery systems provide a flexible framework in which different diagnostic agent types could be effectively transported. These nanostructures composed of liposomes, polymersomes, nanoparticles such as gold nanoparticles and peptide-based vesicles have potential therapeutic properties that are essential for the development of effective nanomedicines [11–14]. In addition, it is well reported that nanomedicine formulations such as extracellular vesicles might improve the activity of antiviral medicines. The fate of such encapsulated drugs may also be affected by nanoparticles, which allow controlled release kinetics, enhanced bioavailability, improved pharmacokinetics, reduced side effects and maximal patient compliance [15].

Furthermore, the unique physicochemical properties of nanocarriers could assist in targeting specific sites that could enable interaction with viral structures. Nanomedicines are also reported to possess the ability to enhance the antiviral therapeutic index [16,17]. To introduce this innovative approach at the clinical level, certain factors namely quality, impact on health and manufacturing issues have to be carefully evaluated [16]. In the medical sector, the applications of vesicular-delivery systems will prove to be very promising, especially in the development of new therapeutic and diagnostic approaches to COVID-19 [18]. Theranostics with vesicular-delivery systems can further offer innovative solutions to combat future coronavirus outbreak.

Currently, there are no specific antiviral treatments that are available for COVID-19. However, previously developed medicines for the treatment of other viral infections along with several anti-malarial drugs are being tested for their efficacy against COVID-19 virus. As stated earlier, clinical studies are underway to determine the effectiveness and safety of several drugs such as chloroquine, arbidol, remdesivir and favipiravir [19]. As a futuristic approach, these drugs can be used with vesicular-delivery systems with a theranostic strategy in developing novel COVID-19 treatment regimens.

## Conclusion

As the epidemic continues to spread, researchers worldwide are actively researching on drugs that could be successful in the battle against COVID-19. Currently, there are no clinically confirmed antiviral treatment options. However, several drugs are now being clinically tested like chloroquine, arbidol, remdesivir and favipiravir that can be targeted with the application of theranostics embedded into vesicular-delivery systems. The effectiveness, preciseness and safety of such advanced technologies have been documented before, nevertheless, further preclinical and clinical trials would validate the applications of these techniques in the treatment of COVID-19.
